# The Association Between Cognitive Performance and Speech-in-Noise Perception for Adult Listeners: A Systematic Literature Review and Meta-Analysis

**DOI:** 10.1177/2331216517744675

**Published:** 2017-12-14

**Authors:** Adam Dryden, Harriet A. Allen, Helen Henshaw, Antje Heinrich

**Affiliations:** 1 Medical Research Council Institute of Hearing Research, 170718 School of Medicine, University of Nottingham, UK; 2 School of Psychology, University of Nottingham, UK; 3 National Institute for Health Research Nottingham Biomedical Research Centre, School of Medicine, University of Nottingham, UK; 4 Otology and Hearing Group, Division of Clinical Neuroscience, School of Medicine, University of Nottingham, UK

**Keywords:** speech perception, cognition, working memory, executive function, hearing loss

## Abstract

Published studies assessing the association between cognitive performance and speech-in-noise (SiN) perception examine different aspects of each, test different listeners, and often report quite variable associations. By examining the published evidence base using a systematic approach, we aim to identify robust patterns across studies and highlight any remaining gaps in knowledge. We limit our assessment to adult unaided listeners with audiometric profiles ranging from normal hearing to moderate hearing loss. A total of 253 articles were independently assessed by two researchers, with 25 meeting the criteria for inclusion. Included articles assessed cognitive measures of attention, memory, executive function, IQ, and processing speed. SiN measures varied by target (phonemes or syllables, words, and sentences) and masker type (unmodulated noise, modulated noise, >2-talker babble, and ≤2-talker babble. The overall association between cognitive performance and SiN perception was *r* = .31. For component cognitive domains, the association with (pooled) SiN perception was as follows: processing speed (*r* = .39), inhibitory control (*r* = .34), working memory (*r* = .28), episodic memory (*r* = .26), and crystallized IQ (*r* = .18). Similar associations were shown for the different speech target and masker types. This review suggests a general association of *r*≈.3 between cognitive performance and speech perception, although some variability in association appeared to exist depending on cognitive domain and SiN target or masker assessed. Where assessed, degree of unaided hearing loss did not play a major moderating role. We identify a number of cognitive performance and SiN perception combinations that have not been tested and whose future investigation would enable further fine-grained analyses of these relationships.

## Introduction

Following a conversation in a noisy environment is difficult, and the effort required increases with hearing impairment (Zekveld, Kramer, & Festen, 2011). Hearing loss (HL) has been extensively investigated as a primary underlying factor for difficulties in speech perception under adverse listening conditions (Agus, Akeroyd, Gatehouse, & Warden, 2009; Humes & Roberts, 1990; Jerger, Jerger, & Pirozzolo, 1991; Smoorenburg, 1992). While HL does explain some of the difficulties, it has also become clear that it cannot be the only driving factor given the following observations: First, listeners with similar auditory sensitivity can differ greatly in their speech-in-noise (SiN) performance (Anderson, Parbery-Clark, Yi, & Kraus, 2011; Vermiglio, Soli, Freed, & Fisher, 2012); second, SiN difficulties can be found in the absence of HL (Gordon-Salant & Fitzgibbons, 1993; Gosselin & Gagne, 2011; Plack, Barker, & Prendergast, 2014); and third, SiN listening difficulties can persist even when HL has been alleviated by hearing aids (Humes, 2002; Studebaker, Sherbecoe, McDaniel, & Gwaltney, 1999). Another factor that has repeatedly been suggested to play a role in SiN perception is cognition (Roberts & Allen, 2016). While investigations of the association between cognitive performance and SiN perception have a long tradition (Pichora-Fuller, Schneider, & Daneman, 1995; Rabbitt, 1968; Tun & Wingfield, 1999; van Rooij & Plomp, 1990, 1992), interest and publications in the field have surged in the past 20 years, leading to the coining of *cognitive hearing science* as a term for the field (Arlinger, Lunner, Lyxell, & Pichora-Fuller, 2009; Rönnberg, Rudner, Lunner, & Zekveld, 2010; Tun, Williams, Small, & Hafter, 2012).

Despite increasing interest in the association between cognitive performance and SiN perception, the emerging picture is far from clear. Not only do measures of SiN perception and cognitive tasks vary greatly across published studies but also research participant samples vary widely and can include any combination of young and old listeners with or without HL, tested under aided or unaided listening.

One way of dealing with the great variability in the field is to use a descriptive approach when summarizing results across studies. This strategy was adopted by Akeroyd (2008) in a review that explored the relationship between individual differences in cognition and SiN perception in normal and hearing-impaired adult listeners (including aided listeners) across 20 studies. He found inconsistencies between study results not only for cases where SiN listening situations and cognitive domains assessed varied across studies but also for cases where the assessed cognitive domain, such as working memory (WM), was constant and only the SiN listening situation varied. Specifically, when surveying all published associations between WM performance and any SiN perception task, Akeroyd found that just over half of the associations (53 of 87) were statistically significant. He concluded that most of these significant associations were shown for studies using SiN perception tests with a sentence (compared to single words) as target speech signal and modulated noise (compared to static noise) background masker.

In a more recent review and meta-analysis, Füllgrabe and Rosen (2016) focused on a single cognitive ability, WM (as measured by the Reading span test), and investigated its association with SiN listening in normal hearing adult listeners. Using a meta-analysis, they examined the association between the performance on the Reading span test and SiN perception using tests with a sentence target presented in colocated background noise. Comparing 24 correlations from 16 studies, they found an overall (nonsignificant) association of .12. As a result of their meta-analysis, the authors suggested that WM contributes relatively little to individual differences in SiN perception in normally hearing younger adult (≤40 years of age) listeners.

The different findings of these two prior reviews may simply be due to differences in the populations studied. The association between WM and SiN perception may not be as ubiquitous as sometimes assumed but instead may vary substantially by age or hearing status of the listener. Alternatively, it is possible that the differences arose because Füllgrabe and Rosen (2016) restricted their search to a single cognitive domain (WM), assessed using one measure (Reading span test).

In this review, we explore both possibilities. First, we consider a range of hearing abilities (normal hearing to moderate HL) in preclinical unaided listeners. Second, we extend the investigation to cognitive abilities other than WM and include a range of measures for each cognitive ability. We systematize all cognitive measures used in the reviewed studies into cognitive domains and subdomains based on well-established cognitive theories. We also systematize SiN measures based on the target speech signal and background masker type. These categorizations enable us to investigate the specific associations between cognitive domain and SiN perception task and how this might contribute to the variability of previously found results.

In contrast to the previous reviews, we hope that our systematic approach will enable us to identify similarities between published studies that use tests assessing the same cognitive domain and similar SiN perception tests and uncover differences between studies that assess different cognitive domains or SiN perception tests. We also aim to highlight any gaps in the published literature by identifying understudied combinations of SiN measures and cognitive domains that warrant further investigation.

Here, our specific research question is the following:

What is the association between cognitive performance and SiN perception for adult listeners with a range of (un-aided) hearing thresholds from normal hearing to moderate hearing loss and does this association vary depending on the type of (cognitive/SiN) measure(s) used?

## Methods

### Categorizing SiN Tests

SiN perception tests can vary on foreground signal, background signal, type of response (open and closed set), signal-to-noise ratios (SNRs) or intelligibility levels, adaptive and nonadaptive paradigms, and signal presentation (headphones or free field) to name but a few aspects. Each of these variations could impact on the manner or extent to which cognitive resources are required to perceive the speech message. As we cannot consider all aspects in this review, we will focus on the examination of the role that foreground and background signals might play for the association between cognition and SiN perception. By systematizing SiN measures based on the foreground (target) and background (masker, i.e., the noise) signals, we can investigate whether all SiN measures within the same category of foreground or background sound show a similar relationship with a particular cognitive measure.

We categorize the foreground target according to its lexical complexity from simplest to most complex into (a) phonemes and syllables, (b) words, and (c) sentences. We classify the target signal as the speech signal that the listener is instructed to respond to. This includes instances where, for example, a phoneme or word target is embedded in a more complex signal such as a sentence or a carrier phrase. When a participant is instructed to repeat a full sentence, but unbeknownst to them the response is scored only on the final word, this will be classified as a sentence target signal. This is because the task, not the scoring, defines the characteristics of the signal. There were no reported instances of participants’ being aware of the scoring procedure for any SiN perception test in the included studies.

We chose lexical complexity as the basis for categorization because it has been shown to be important for the manner or extent to which cognitive processes are engaged (Heinrich, Henshaw, & Ferguson, 2015, 2016; Heinrich & Knight, 2016; Xu et al., 2005). For example, when measuring correlations between cognition and SiN perception, Heinrich and Knight (2016) showed an increased association between the Reading span test and the Letter–Number Substitution tests when comparing words and sentences, respectively, in a background of speech-modulated noise. Moreover, in a language comprehension fMRI study, Xu, Kemeny, Park, Frattali, and Braun (2005) mapped brain activation in a single word and sentence comprehension. They found increased activation in regions including Broca’s area, left middle temporal gyri, right posterior cerebellum, left putamen, and ventral thalamus for sentence compared to single word, comprehension, indicating a differing network of activation for these types of stimuli.

We conceptualize differences in the background signal by considering the extent to which the background engages energetic and informational masking. Energetic masking refers to a masking signal that physically obscures a target signal and where the interference to the target is due to the physical overlap with the background signal (Kidd, Mason, Deliwala, Woods, & Colburn, 1994). Informational masking on the other hand refers to a masking signal that contains intelligible sounds, such as words and phonemes, and where the interference to the target is due to the distracting quality of the masker (Pollack, 1975).

Placing background signals on a continuum between energetic and informational masking resulted in the following order of (decreasing) energetic and (increasing) informational masking: (a) unmodulated noise, (b) modulated noise, (c) multiple (>2) background talkers, and (d) a single- or two-distractor voice(s). Background signals with one- and two-distractor voices were separated in this classification from multiple background voices for two reasons. First, Simpson and Cooke (2005) showed that the difference in intelligibility of foreground speech is particularly marked for one- and two-background talker(s) versus a higher number of talkers. Second, it has been suggested that increased intelligibility of background sounds (indicating increased informational masking) engages cognitive processes such as inhibitory control and attention (Mattys, Brooks, & Cooke, 2009) that help to disentangle the target signal from the masker (Freyman, Balakrishnan, & Helfer, 2004). Possibly, these processes are not engaged to the same extent by multiple background voices.

**Figure 1.**
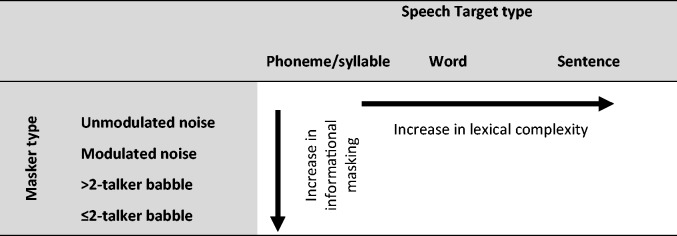
Speech-in-noise test matrix displaying the categories for classifying speech target and masker type. >2-talker babble: speech babble consisting of more than two speakers; ≤2-talker babble: speech “babble” containing two or only one distractor voice.

The matrix for the categorization of the SiN perception tests used in the studies considered in this review is displayed in Figure 1. Within these categories, intelligibility levels, adaptive versus nonadaptive paradigms, and signal presentation are not distinguished. We recognize this as a limitation of our categorization system. However, due to the vast heterogeneity in SiN perception tests in previous studies, some simplification was necessary, and we chose to investigate the role of foreground and background signals for this review while generalizing over all other differences.

### Categorizing Cognitive Measures

Cognitive function associated with SiN perception has been assessed using a wide variety of measures. This can make the direct comparison between studies difficult. We address this issue by abstracting from a particular cognitive test to the tested cognitive domain and subdomain being assessed. In total, we distinguish five cognitive domains (attention, executive processes, memory, intelligence, and processing speed) and nine cognitive subdomains (alerting, orienting, set-shifting, inhibitory control, WM, episodic memory, fluid and crystallized intelligence, and processing speed) based on contemporary cognitive theories (Baddeley, 2000; Diamond, 2013; Miyake et al., 2000; Petersen & Posner, 2012; Salthouse, 2000).

We define each domain and its constituting subdomains below and briefly explain their proposed involvement in SiN perception. Although we recognize that an individual test can load on multiple cognitive domains (Surprenant & Neath, 2009), for the purpose of this review, we categorize each test only according to the main subdomain it is theorized to assess. We categorize cognitive performance at the level of subdomain for two main reasons. First, this level specificity allows us to differentiate specific subdomains of interest for SiN perception. For example, assessing set-shifting, WM, and inhibitory control as individual subdomains of executive control may be of added value and interest compared to the consideration of a single executive process domain. Second, by categorizing cognitive performance at the level of subdomain, we hope to reduce heterogeneity within each domain.

Supplementary Table 1 provides a full list and description of all cognitive tests used in the reviewed studies, ordered by cognitive domain and subdomain. Please note that a few tests, such as the Text reception threshold (Zekveld, George, Kramer, Goverts, & Houtgast, 2007), which is the theorized visual equivalent to the Speech reception threshold test, are not included in this review because they are not readily definable within our single cognitive domain framework.

One limitation to highlight is that we did not account for differences in measurement or scoring methods across cognitive tests that assess a single subdomain. Although we recognize its importance, this is not a factor we were able to specifically assess in this review. For a review on general method test bias in psychometric tests, see Podsakoff, MacKenzie, Lee, and Podsakoff (2003), and for an overview on memory span tasks, see Conway et al. (2005).

#### Attention

We conceptualized tests assessing attention within Posner and Petersen’s (1990) framework, which considers three distinct but interconnected processes: (a) alerting, (b) orienting, and (c) executive control. Given the central role that executive control is assumed to play for SiN perception (Pichora-Fuller et al., 2016; Tamati, Gilbert, & Pisoni, 2013; Zekveld, Rudner, Kramer, Lyzenga, & Rönnberg, 2014), we considered the further subdomains of executive processing separately from attention.

##### Alerting

Alerting is the ability to prepare and sustain attention to a high priority signal (Posner & Petersen, 1990). It may be important for SiN perception because it allows listeners to focus on the speech target in an environment of other noise sources (Binder et al., 1994; Heald & Nusbaum, 2014). It is possible that it plays a particularly important role for more complex target signals (such as whole sentences) because they require sustained attention for a longer period of time.

##### Orienting

Orienting refers to the ability to, overtly or covertly, prioritize sensory input from a particular spatial or temporal location or modality (Posner & Petersen, 1990). It may be important for SiN perception, particularly in situations of spatial separation because it allows temporal and spatial preferential selection of a target signal (Astheimer & Sanders, 2009; Calvert, Brammer, & Iversen, 1998).

#### Executive processes

Executive processes control and coordinate performance of complex cognitive tasks. They are closely related to attention and are sometimes considered as one of its subdomains (Posner & Petersen, 1990). Due to their potential importance for SiN perception, we considered them as a separate domain and subdivided them further based on Miyake et al. (2000) into three subdomains: (a) set-shifting, (b) inhibitory control, and (c) updating (termed “WM” in the context of this review).

*Set-shifting* refers to the ability to switch between tasks, operations, or mental sets (Miyake et al., 2000). Set-shifting ability is thought to be closely related to representations of internal speech and task-specific organization (Cragg & Nation, 2010). It might also be predicted that it is important when a listener has to shift from one speech target to another.

*Inhibitory control* is a process by which a strong interfering factor is overcome in order to maintain focus on the desired target or task (Diamond, 2013; Hasher & Zacks, 1979). Inhibitory control has been suggested to play a role for SiN perception in several ways. First, poor inhibition may increase susceptibility to background noise during SiN perception, particularly in informational masking conditions (Janse, 2012). Second, poor inhibition may make it harder for listeners to successfully select the target during lexical access (Sommers & Danielson, 1999). Third, inhibition may have a general role in degraded signal restoration (Janse & Jesse, 2014; Mattys, Davis, Bradlow, & Scott, 2012).

*WM* is a limited-capacity process by which we simultaneously store, process, and manipulate information necessary to complete complex tasks (Daneman & Carpenter, 1980). Prominent WM theories include the multicomponent model proposed by Baddeley and Hitch (Baddeley, 2000; Baddeley & Hitch, 1974) and the activation model by Engle and Kane (2004). Both models propose a single amodal executive processing component required for a task-driven focus of attention. In addition, Baddeley (2000) also proposed amodal and modality-specific separate slave systems for information storage. The concept of WM is very prominent in the SiN perception literature. It has been incorporated into a prominent framework on the involvement of cognition in speech perception, the Ease of Language Understanding model (Rönnberg, 2003; Rönnberg et al., 2013; Rönnberg, Rudner, Foo, & Lunner, 2008). The Ease of Language Understanding model posits that WM plays a role in the restoration of degraded speech signals and in the *inhibition* of masking signals (Rönnberg et al., 2013). However, whether WM is equally important for all groups of listeners or only for those with a degraded input (e.g., listeners with hearing impairment) is a matter of considerable debate. For a task to be classed as WM within this review, it had to contain both a storage and a manipulation component. The type of information (verbal or nonverbal) and the modality of presentation (auditory or visual) were of no relevance here.

#### Memory

Memory is the faculty by which information is encoded, stored, and retrieved (Atkinson & Shiffrin, 1968). There are many classifications of memory depending on the aspect of memory that is emphasized. Here, we are particularly interested in *episodic memory*, which according to Tulving (1972) refers to the encoding of distinct episodes of information for later recall. The distinguishing feature of episodic memory compared with WM for the purpose of the current review is the presence (WM) or absence (episodic memory) of a manipulation component. Episodic memory has been hypothesized to be important for SiN perception because with longer speech signals a listener has to hold a speech trace in mind in order to integrate it with previously heard or retrieved information (Goldinger, 1996; Rönnberg et al., 2008).

#### Intelligence

General intelligence refers to the overall mental ability common to performance of all cognitive tasks (Spearman, 1904). Cattell (1963) differentiates between fluid and crystallized intelligence.

*Fluid intelligence* refers to the general ability to solve problems and use abstract reasoning. It may be related to SiN perception through its link with WM and executive control and may be particularly important in complex listening situations such as dichotic listening (Engle, 2002; Meister et al., 2013b). Fluid intelligence is typically assessed using nonverbal tasks.

*Crystallized intelligence* refers to language- and culture-specific knowledge and skills, which are acquired over time. It is thought to be important for SiN perception when the listening task requires increased reliance on lexical or general knowledge. Such situations may arise when the masker is informational or when target stimuli contain substantial contextual support (Schneider, Avivi-Reich, & Daneman, 2016).

#### Processing speed

*Processing speed* is the rate at which information is processed in order to execute a task. It has been suggested to play a crucial role in explaining age-related changes in cognition (Salthouse, 2000). Processing speed has been implicated in speech perception due to the sequential nature of the speech signal, which requires rapid and repeated recruitment of other cognitive processes such as, but not limited to, working and episodic memory and linguistic knowledge (Wingfield, 1996). It could be speculated that such rapid comprehensive processing is even more important when the speech is complex (e.g., long complex sentences, fast speech rate, and large number of propositions) or the speech signal is degraded. In this case, the speed with which this knowledge can be accessed determines how deeply the speech is processed and how much extra load is placed on memory processes (Gordon-Salant & Fitzgibbons, 2001; Wingfield, Tun, Koh, & Rosen, 1999). Older adults tend to process information at a slower speed, so it may well be that slowing processing speed is a factor for declining SiN perception in older listeners (Pichora-Fuller, 2003).

### Review Guidelines

Although this is a review of basic research, the conduct and reporting of this systematic review and meta-analysis was informed by health-care systematic review guidelines, including the Centre for Research and Dissemination’s (2009) guidance for undertaking reviews in health care, the Grading Quality of Evidence and Strength of Recommendations (Atkins et al., 2004), and the Preferred Reporting Items for Systematic Reviews and Meta-Analyses checklist (Moher, Liberati, Tetzlaff, Altman, & PRISMA Group, 2009).

### Systematic Search Strategy and Study Identification

This review will consider all of the existing literature published to May 2017. Only published studies appearing in peer-reviewed journals were considered. The literature search was conducted using Web of Science, PubMed, and Scopus. The search terms “speech” AND “cognit*” AND “noise” OR “babble” OR “talker” NOT “children” NOT “imaging” were entered across all categories and yielded 19,012 hits. The removal of duplicate studies reduced this number to 18,764 studies.

### PICOS Screening Criteria

In the screening process, each of the 18,764 studies were assessed, by reading the titles and abstracts, and included or eliminated based on the PICOS (Population, Intervention, Comparator, Outcome, Study design) criteria (Centre for Research and Dissemination, 2009). Studies which could not be assessed by the titles and abstracts were subject to a full-text search. A. D. and H. H. independently conducted the screening and identification processes. In the full-text search, A. D. collated, removing any duplication, the studies selected in the identification.

#### Population

Studies reporting results of at least one group of adults (18+ years) with

- hearing in the range of normal sensitivity to moderate HL measured using pure-tone audiometry (pure-tone average thresholds better than 71 dB HL across at least three octave frequencies below 8 kHz)
- no reported previous or current hearing intervention and excluding studies which are explicit in reporting listener groups, which include
- Non-native speakers
- Visual impairment not corrected to normal
- Diagnoses of neurological or psychiatric comorbidities.

#### Intervention

A minimum of one audio-only SiN perception measure consisting of a concurrently and colocally presented target and masker. A composite SiN outcome measure is only accepted if the individual measures that make up the composite assess target or masker combinations within the same category as defined earlier, for example, a composite comprising two or more individual measures of sentence in 4-talker babble.

#### Comparator

A minimum of one cognitive ability measure. A composite was only accepted if the individual measures that made up the composite assessed a single cognitive subdomain (see Categorizing of Studies section). Note, any cognitive test that was conducted as part of a dual-task paradigm (e.g., in competing noise) was not considered.

#### Outcome

A quantitative comparison between SiN intelligibility and cognitive measures (either correlation, regression, or linear model analyses).

#### Study design

Single time point association studies (or single time point associations taken from a larger study) were considered. SiN intelligibility measures could be presented within either an adaptive or a fixed SNR procedure across the entire intelligibility range. Other measures, for example, reaction times, were not considered here. Both the SiN perception and cognitive performance measures were required to have been conducted in a quiet room free from distraction, and not as part of a brain imaging paradigm. Only data collected from participants individually where considered. Data collected as part of a group testing session where not included.

### Screening Results

**Figure 2.**
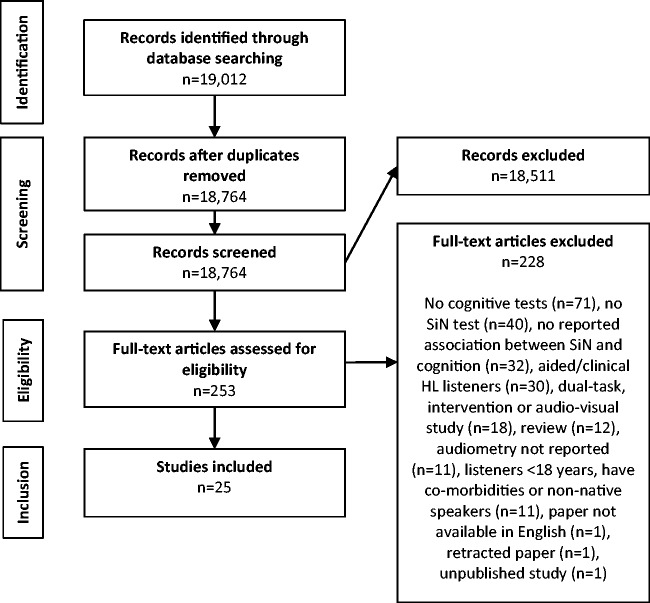
PRISMA flow chart of literature search showing the identification, screening, eligibility, and inclusion phases of the search.

After initial abstract and title screening, a full-text assessment was deemed necessary for 253 studies. This process resulted in a final set of 25 articles eligible for inclusion in the review. None of the articles included in the review reported more than one study; hence, the number of articles equaled the number of included studies. Figure 2 shows a flow diagram of each stage of the search process. Only one study (Zekveld et al., 2011) included a group with hearing aid intervention, alongside a group with hearing thresholds ranging from normal hearing to untreated moderate HL. In this case, only the data from the untreated HL group were included in the review. In all other cases, any participant HL was untreated. While the hearing level of listeners in all remaining studies was described as normal or age-normal, the range of pure-tone averages was considerable across studies.

### Assessment of Risk of Study Bias

We devised a risk of bias assessment on which each of the 25 full-text articles included in the review were assessed. This scoring system was informed by risk of bias assessments for clinical trials (Higgins et al., 2011). Although only the universal criteria were retained, we must be aware that the reporting requirements of experimental studies are not as rigorous as clinical trials, and so we may not expect them to report to these standards.

Supplementary Table 2 details the four questions of the risk of bias assessment (2a) and the score key (2b). All 25 studies were scored by H. A. A. In addition, all of the studies were also independently scored by one of the other coauthors. Studies whose scores diverged in more than one category were discussed between scorers until a consensus was reached on at least three of the four questions. If a divergence remained in one question for a given study, the maximum divergence allowed was one point.

### Categorization of Studies

Each study’s methods were read, and the SiN and cognitive measures were categorized according to the matrix in Figure 1 and cognitive measures according to Supplementary Table 1.

### Categorization Based on Participant Groups

As it has been suggested that HL may play a moderating role in the association between cognitive performance and SiN perception (Füllgrabe & Rosen, 2016), we consider, where possible, the association of cognitive performance and SiN perception for studies where listeners’ hearing ability ranged from normal hearing to mild HL and where ability ranged from normal hearing to moderate HL. Unfortunately, it was not possible to assess associations across the categories (normal hearing, mild HL, and moderate HL) independently due to the overlapping sampling methods employed by the studies included in this review. If the association between cognitive performance and SiN listening is universal, then we would expect the inclusion or exclusion of listeners with moderate HL not to make an appreciable difference to the strength of association. If on the other hand HL moderates the relationship, then we might expect the level of association to change depending on the presence of the listeners with moderate HL. Such a differentiation needs to be balanced against the fact that the number of reviewed studies is rather small and the combination of SiN and cognitive conditions rather larger. Hence, in cases where too few studies reported a particular combination of SiN and cognitive measures, hearing range was not differentiated.

We categorized reported audiometric thresholds according to BSA (2011) guidelines in normal hearing (<20 dB HL average across octave frequencies, 0.25–4 kHz), mild HL (20–40 dB HL, 0.25–4 kHz), and moderate HL (41–70 dB HL, 0.25–4 kHz). We then categorized studies according to their participant group. Sixteen studies fitted into the normal hearing to mild HL category, and nine into the normal hearing to moderate HL category.

Here we only considered preclinical, unaided listeners. Hearing intervention and aided listening may influence the association between cognitive performance and the processing of incoming (altered) acoustic signals (Ferguson et al., 2017). For a review investigating the role of cognitive subdomains in hearing intervention or impairment, see Taljaard, Olaithe, Brennan-Jones, Eikelboom, and Bucks (2016).

### Meta-Analyses

In order for a meta-analysis to be performed for a given cognition and SiN measure association a minimum of four studies was required. This number was chosen to provide a balance between calculating as many meta-analyses as possible while also maintaining a minimum of statistical power. For all meta-analyses, if more than one quantitative comparison was reported in a single study (e.g., the same SiN measure correlated with two different measures of WM), the mean value was computed from the multiple correlation coefficients.

Meta-analyses and Forest plots were computed using MedCalc® version 16.8.4. A random-effects model was chosen for the calculation of pooled associations because it incorporates random variation both within and between studies. The applied model calculated weighted summaries of individual correlations based on the Hedges and Olkin (1985) method. Heterogeneity between studies was assessed using the *I*^2^ statistic (Higgins & Thompson, 2002) with 0% showing no heterogeneity between studies and a higher percentage value indicating higher heterogeneity between studies included in the pooled association. No comparison was removed on the basis of high heterogeneity. Forest plots aid the comparison of individual studies included in the meta-analysis. Within each Forest plot, marker size varies according to weight assigned to each study based on the random-effects model. Larger symbols indicate a larger contribution to the pooled (or average) associations.

## Results

### Included Studies

A summary of each of the 25 articles included in the review is given in Supplementary Table 3. The table includes demographic information about participants and categorizations of SiN and cognitive measures for each study.

### Risk of Bias Assessment

The results of the bias assessment are displayed in Table 1. Risk of bias was high for Q1 as the majority of these basic investigations did not include a sample size calculation to inform statistical power. For those studies that excluded participant data, adequate justification was provided in most cases (Q2). Around a third of studies did not provide sufficient information to confirm that results were reported for all included outcome measures (Q3). The majority of studies did not report any conflicts of interest (Q4). Taken together, although we can be relatively confident that the reported results are at low risk of reporting bias, we are unable to confirm whether the individual studies included in this review and meta-analysis include sample sizes that are sufficient to adequately detect statistically significant associations. One motivation to conduct a meta-analysis is to overcome this shortcoming.

**Table 1.** Bias Scores for Each Article Included in the Review.

Study	Q1: Sample size	Q2: Exclusions	Q3: Outcomes	Q4: Conflicts
Anderson et al. (2013)	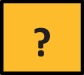	N/A	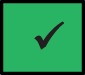	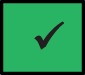
Besser et al. (2012)	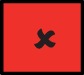	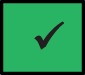	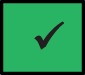	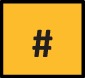
Carroll et al. (2016)	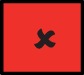	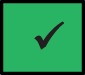	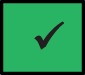	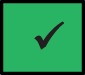
Cervera et al. (2009)	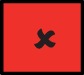	N/A	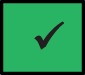	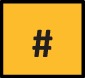
Ellis and Rönnberg (2014)	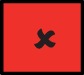	N/A	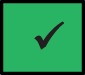	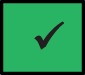
Gordon-Salant et al. (2015)	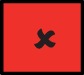	N/A	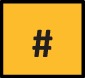	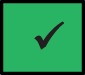
Gordon-Salant & Cole (2016)	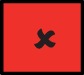	N/A	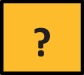	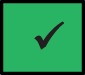
Heinrich et al. (2015)	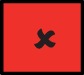	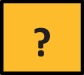	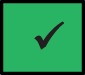	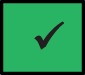
Heinrich and Knight (2016)	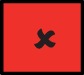	N/A	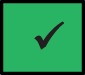	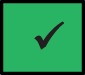
Helfer and Freyman (2014)	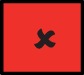	N/A	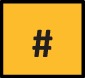	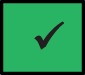
Janse (2012)	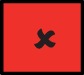	N/A	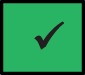	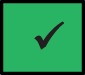
Koelewijn et al. (2012)	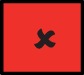	N/A	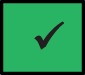	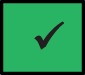
Meister et al. (2013a)	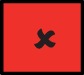	N/A	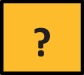	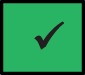
Meister et al. (2013b)	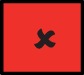	N/A	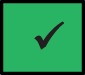	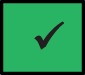
Parbery-Clark et al. (2009)	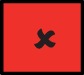	N/A	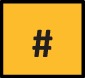	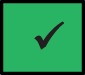
Parbery-Clark et al. (2011)	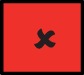	N/A	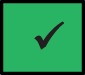	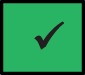
Rönnberg et al. (2014)	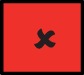	N/A	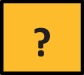	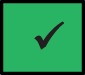
Slater and Kraus (2016)	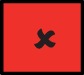	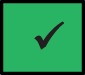	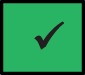	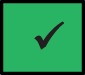
Stenbäck et al. (2015)	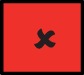	N/A	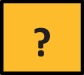	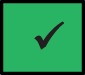
Surprenant (2007)	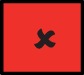	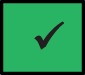	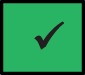	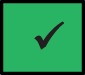
Tun and Wingfield (1999)	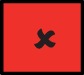	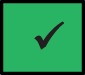	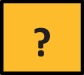	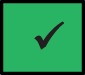
Uslar et al. (2013)	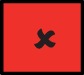	N/A	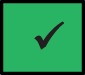	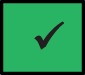
Veneman et al. (2013)	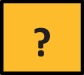	N/A	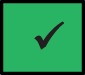	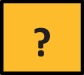
Zekveld et al. (2011)	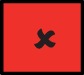	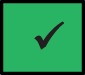	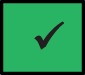	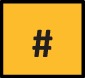
Zekveld et al. (2014)	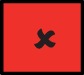	N/A	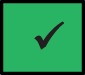	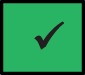

*Note*. Full details of the scoring questions and verbal descriptions of the response categories are in Supplementary Table 2. Briefly Q1: Did the authors include a sample size justification? Q2: If any participant data is excluded from the analysis is a clear justification given? Q3: Were all the outcome measures in the methods included in the results? Q4: Were there any conflicts of interest? That is, was the study funded or conducted by a body with vested interests in the results? Scores highlighted in red indicate a high risk of bias, scores in green indicate low risk of bias, and scores in orange indicate an unknown risk of bias. For each question, the score could be ✗ (Q1–3 Insufficient information for judgement/Q4. Clear conflict of interest) ? (Q1–3 Incomplete information/Q4 unclear), ✓ (Q1–3 Appropriate use and sufficient information/Q4 no conflict of interest) or N/A for Q2 (i.e., there were no relevant instances). Where there was a difference between the scorers this can be seen by the total being a # and was considered the equivalent risk as ?

### SiN Measures

The 25 studies tested a total of 1,026 listeners on a total of eight different combinations of foreground (target) and background (masker) signals. Table 2 shows the frequencies with which each target–masker combination was used. Relatively few studies used phonemes or words as speech target stimuli. Of those that used sentences, all types of masker were used, with unmodulated noise being the most frequent.

**Table 2.** Frequency of Target and Masker Combinations Across All 25 Reviewed Studies.

	Speech target type			
Masker type	Phoneme/ syllable	Word	Sentence	*Total*
Unmodulated noise	2	0	13	*15*
Modulated noise	0	1	5	*6*
>2-talker babble	0	3	10	*13*
≤2-talker babble	1	*0*	5	*6*
*Total*	*3*	*4*	*33*	*40*

*Note.* Where target or masker type combinations are repeated within a study, the combination is only recorded once.

### Cognitive Measures

The 25 studies included a total of 59 cognitive measures which comprised 2 measures of alerting, 1 of orientating, 2 of set-shifting, 7 of inhibitory control, 26 of WM, 7 of episodic memory, 2 of fluid intelligence, 8 of crystallized intelligence, and 4 measures of processing speed.

### Meta-Analyses

In total, we carried out five sets of meta-analyses (reported in Tables 3 to 7). In the first set of analyses, the overall association between all cognitive performance (collapsed across all subdomains) and SiN categories (collapsed across all categories) was investigated. It was carried out with a subanalysis for the groups with different amounts of HL. A second set of analyses looked at each cognitive subdomain in turn with SiN measures collapsed across all categories. Subanalyses were conducted for the two HL groups where possible.

**Table 3.** Meta-Analysis of the Association Between Cognition (All Subdomains Collapsed) and SiN Perception (All Conditions Collapsed) for All Listeners, and Subdivided for Ranges “Normal Hearing to Mild HL” and “Normal Hearing to Moderate HL.”

Cognitive subdomain	Target speech	Masker	Hearing range	Pooled sample size	Pooled association (*r*)	95% CI of *r*	*Z* statistic and *p* value	*I* ^2^	95% CI of *I*^2^	No. of sign studies/No. of all studies
Collapsed	Collapsed	Collapsed	All	1026	.31	[0.23, 0.39]	7.2, <.001	44%	[9.2, 64.8]	12/25
			Normal hearing to mild HL	595	.31	[0.20, 0.42]	5.28, <.001	47%	[5.7, 70.5]	8/16
			Normal hearing to moderate HL	431	.32	[0.19, 0.43]	4.82, <.001	42%	[0.0, 73.1]	4/9

*Note.* CI = confidence interval; HL = hearing loss; *I*^2 ^= heterogeneity statistic; SiN = speech-in-noise.

For the third and fourth sets of analyses, the SiN measures were separated along the two dimensions of target and masker types, and associations with a particular cognitive subdomain were calculated for each dimension. For instance, when the association with SiN target types was investigated, separate group analyses with cognitive subdomains were calculated for each SiN *target type* (phonemes, words, and sentences) while collapsing over all types of background masker. Similarly, when the association with background masker was investigated, separate group analyses with cognitive subdomain were calculated for each type of masker (unmodulated noise, modulated noise, >2-talker babble, and ≤2-talker babble) while collapsing across all SiN target types. In a final set of analyses, the association between cognitive subdomains and specific SiN perception measures (not collapsing across target or background signals, e.g., *sentences-in-modulated noise*) was assessed.

#### Association between cognitive performance (collapsed across subdomains) and SiN perception (collapsed across all target or masker types)

The analysis of the association between a general measure of cognitive performance and a general measure of SiN perception, when considering the full range of listeners, showed an association of .31. The subanalysis of hearing range showed associations of .31 for listeners with normal hearing to mild HL and .32 for listeners with normal hearing to moderate HL with virtually overlapping confidence intervals.

**Figure 3.**
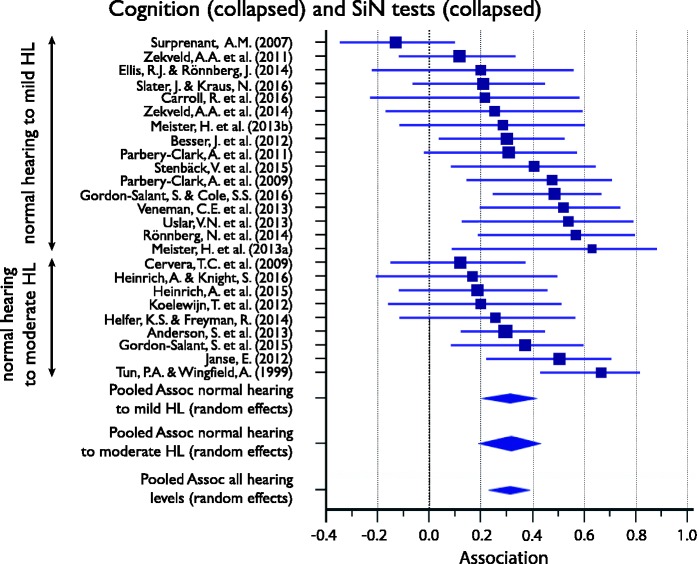
Forest plot showing the association between cognition (all subdomains collapsed) and speech-in-noise (SiN; all conditions collapsed) for listeners with normal hearing to mild and normal hearing to moderate hearing loss. Marker sizes for individual studies (squares) are weighted on random-effect model weights. Whiskers represent 95% confidence interval. Pooled effects, calculated using a random-effects model, are shown as diamonds with the symbols extending to 95% confidence interval.

Table 3 shows the full descriptive statistics of the meta-analysis for the entire group of studies and for the two subgroups of listeners. Figure 3 displays the Forest plots of the individual studies contributing to, as well as the mean association of, each of the three meta-analyses. The plots show that while most associations are positive, only some reach statistical significance.

#### Association between cognitive subdomains and SiN perception (collapsed across all target or masker types)

**Figure 4.**
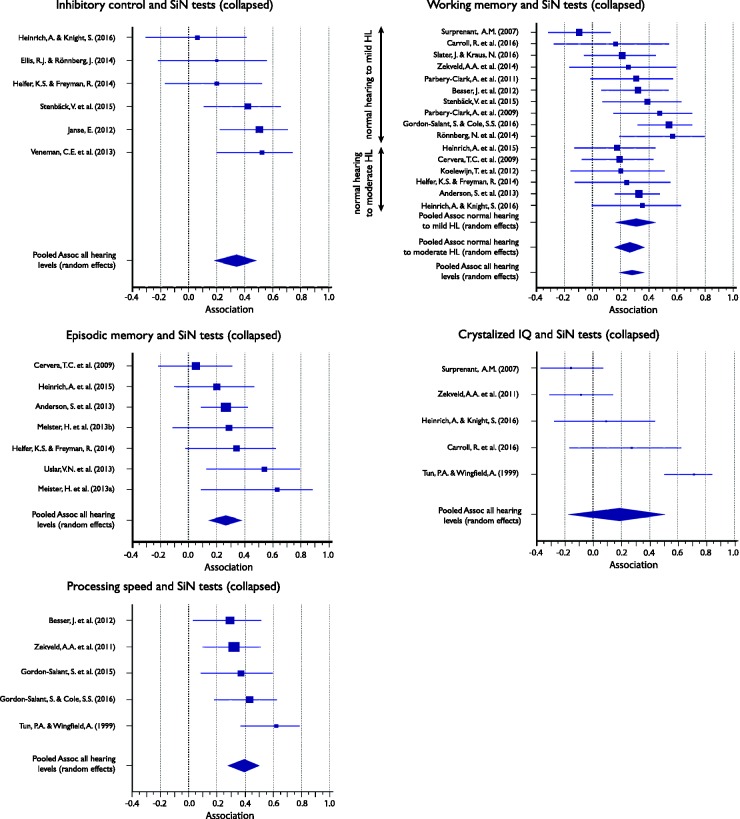
Forest plots showing the association between cognitive subdomain and speech-in-noise (SiN) (all conditions collapsed) for all listeners unless otherwise stated. Marker sizes for individual studies (squares) are weighted on random-effects model weights. Whiskers represent 95% confidence interval. Pooled effects, calculated using a random-effects model, are shown as diamonds with the symbols extending to 95% confidence interval.

Table 4 shows the full descriptive statistics for the association between cognitive subdomain and SiN perception measures, which was computed for inhibitory control, WM, episodic memory, crystallized intelligence, and processing speed. For WM, the meta-analyses were also run separately for groups of listeners whose hearing ranged between normal and mild HL and normal and moderate HL. Associations ranged between .18 and .39 and were significant for all subdomains, except crystallized intelligence. Figure 4 displays the Forest plots of the individual results contributing to, as well as the mean association of, each meta-analysis of the five subdomains. The plots show that while most associations are positive, only some reach statistical significance.

**Table 4.** Meta-Analysis of the Association Between Cognitive Performance Subdomain and SiN Perception Measures (All Target/Masker Conditions Collapsed) for All Listeners, Unless Otherwise Stated.

Cognitive subdomain	Target speech	Masker	Hearing range	Pooled sample size	Pooled association (*r*)	95% CI of *r*	*Z* statistic and *p* value	*I* ^2^	95% CI of *I*^2^	No. of sign studies/No. of all studies
Inhibitory control	Collapsed	Collapsed	All	189	.34	[0.18, 0.48]	4.08, <.001	23%	[0.0, 66.8]	3/6
Working memory			All	720	.28	[0.19, 0.37]	5.89, <.001	34%	[0.0, 63.6]	6/16
Working memory			Normal hearing to mild HL	409	.31	[0.16, 0.45]	3.96, <.001	57%	[12.9, 78.7]	5/10
Working memory			Normal hearing to moderate HL	311	.26	[0.15, 0.37]	4.61, <.001	0%	[0.0, 25.4]	1/6
Episodic memory			All	307	.26	[0.14, 0.38]	4.12, <.001	12%	[0.0, 74.6]	3/7
Crystallized IQ			All	237	.18	[−0.18, 0.50]	1.00, .32	86%	[69.2, 93.6]	1/5
Processing speed			All	263	.39	[0.28, 0.50]	6.14, <.001	11%	[0.0, 82.6]	5/5

*Note.* CI = confidence interval; HL = hearing loss; *I*^2 ^= heterogeneity statistic; SiN = speech-in-noise.

#### Association between cognitive subdomains and SiN target speech types (collapsed across maskers)

**Figure 5.**
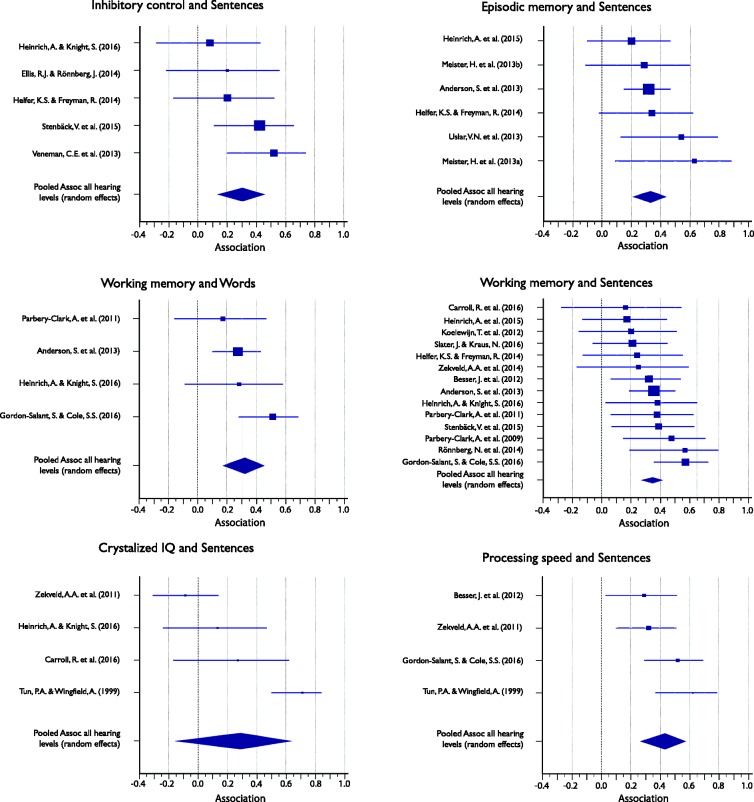
Forest plots showing the association between cognitive subdomains and speech-in-noise target types (collapsed over masker) for all listeners. Marker sizes for individual studies (squares) are weighted on random-effects model weights. Whiskers represent 95% confidence interval. Pooled effects, calculated using a random-effects model, are shown as diamonds with the symbols extending to 95% confidence interval.

Associations ranged between .29 and .43 and were significant for all subdomains, except crystallized intelligence (see Table 5). Figure 5 displays the Forest plots of the individual results contributing to, as well as the mean association of, each of the six meta-analyses. The plots show that while most associations reported by individual studies are positive, only some reach statistical significance.

**Table 5.** Meta-Analysis of the Association Between Cognitive Performance Subdomains and SiN Target Speech Types (Collapsed Across Maskers) for All Listeners.

Cognitive subdomain	Target speech	Masker	Hearing range	Pooled sample size	Pooled association (*r*)	95% CI of *r*	*Z* statistic and *p* value	*I* ^2^	95% CI of *I*^2^	No. of sign studies/No. of all studies
Inhibitory control	Sentences	Collapsed	All	150	.30	[0.13, 0.46]	3.40, .001	11%	[0.0, 82.6]	2/5
Working memory	Words			240	.32	[0.17, 0.45]	4.12, <.001	24%	[0.0, 90.2]	2/4
Working memory	Sentences			590	.34	[0.27, 0.42]	8.37, <.001	0%	[0.0, 47.8]	8/14
Episodic memory	Sentences			252	.33	[0.21, 0.44]	5.23, <.001	0%	[0.0, 64.5]	3/6
Crystallized IQ	Sentences			162	.29	[−0.16, 0.64]	1.27, .205	86%	[66.5, 94.4]	1/4
Processing speed	Sentences			218	.43	[0.27, 0.57]	4.83, <.001	45%	[0.0, 81.8]	4/4

*Note.* CI = confidence interval; *I*^2 ^= heterogeneity statistic; SiN = speech-in-noise.

#### Associations between cognitive subdomains and masker types (collapsed across target speech types)

**Figure 6.**
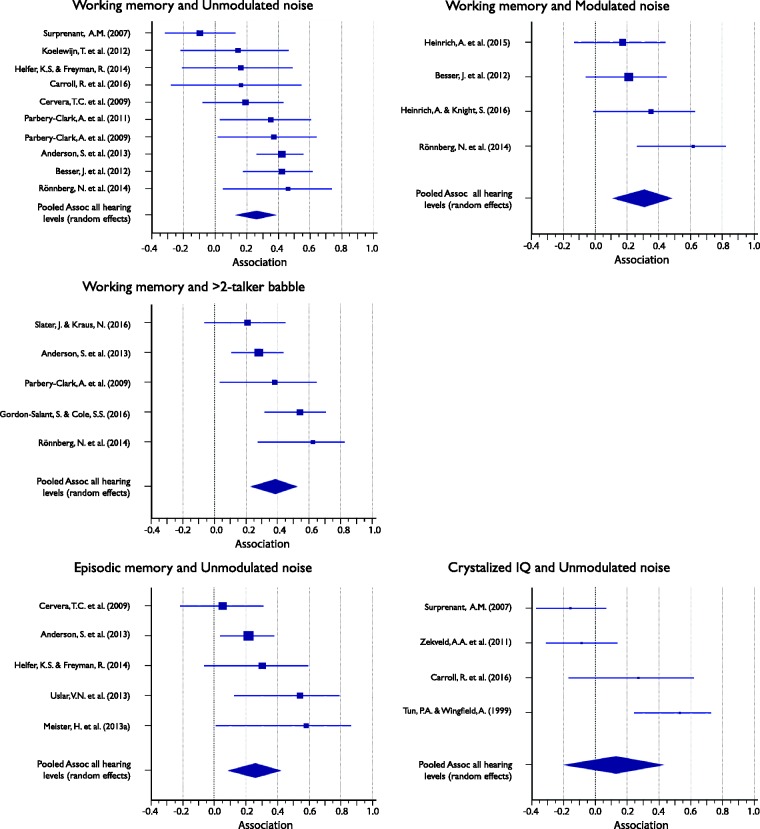
Forest plots showing the association between cognitive subdomains and speech-in-noise (SiN) masker types (collapsed over target) for all listeners. Marker sizes for individual studies (squares) are weighted on random-effects model weights. Whiskers represent 95% confidence interval. Pooled effects, calculated using a random-effects model, are shown as diamonds with the symbols extending to 95% confidence interval.

Associations ranged between .13 and .39 and were significant for all but one (crystallized intelligence) cognitive subdomain (see Table 6). Figure 6 shows the Forest plots of the individual results contributing to, as well as the mean average association of, each of the five meta-analyses. Again, despite overall significant average association and generally positive associations, only some of the individual associations were significant.

**Table 6.** Meta-Analysis of the Association Between Cognitive Performance subdomains and SiN Masker Types (Collapsed Across Target Speech Types) for All Listeners.

Cognitive subdomain	Target speech	SiN masker	Hearing range	Pooled sample size	Pooled association (*r*)	95% CI of *r*	*Z* statistic and *p* value	*I* ^2^	95% CI of *I*^2^	No. of sign studies/No. of all studies
Working memory	Collapsed	Unmodulated noise	All	479	.26	[0.13, 0.38]	3.76, <.001	50%	[0.0, 76.0]	5/10
Working memory		Modulated noise		151	.31	[0.11, 0.48]	3.00, .003	34%	[0.0, 76.5]	1/4
Working memory		>2-talker babble		280	.39	[0.23, 0.52]	4.54, <.001	45%	[0.0, 80.0]	4/5
Episodic memory		Unmodulated noise		237	.26	[0.08, 0.42]	2.88, .004	32%	[0.0, 74.2]	3/5
Crystallized IQ		Unmodulated noise		207	.13	[−0.20, 0.43]	0.75, .45	80%	[47.8, 92.5]	1/4

*Note.* CI = confidence interval; *I*^2 ^= heterogeneity statistic; SiN = speech-in-noise.

**Table 7.** Meta-Analysis of the Association Between Cognitive Performance Subdomains and SiN Target Speech and Masker Types for All Listeners.

Cognitive subdomain	Target speech	Masker	Hearing range	Pooled sample size	Pooled association (*r*)	95% CI of *r*	*Z* statistic and *p* value	*I* ^2^	95% CI of *I*^2^	No. of sign studies/No. of all studies
Working memory	Sentences	Unmodulated noise	All	349	.35	[0.25, 0.44]	6.64, <.001	0%	[0.0, 53.6]	5/8
Working memory	Sentences	Modulated noise		151	.32	[0.12, 0.49]	3.03, .002	36%	[0.0, 77.6]	2/4
Working memory	Sentences	>2-talker babble		317	.43	[0.28, 0.56]	5.21, <.001	50%	[0.0, 80.2]	5/6
Episodic memory	Sentences	Unmodulated noise		182	.31	[0.14, 0.47]	3.44, .001	15%	[0.0, 89.0]	3/4

*Note.* CI = confidence interval; *I*^2 ^= heterogeneity statistic; SiN = speech-in-noise.

#### Associations between cognitive subdomains and specific SiN target speech or masker type combinations

**Figure 7.**
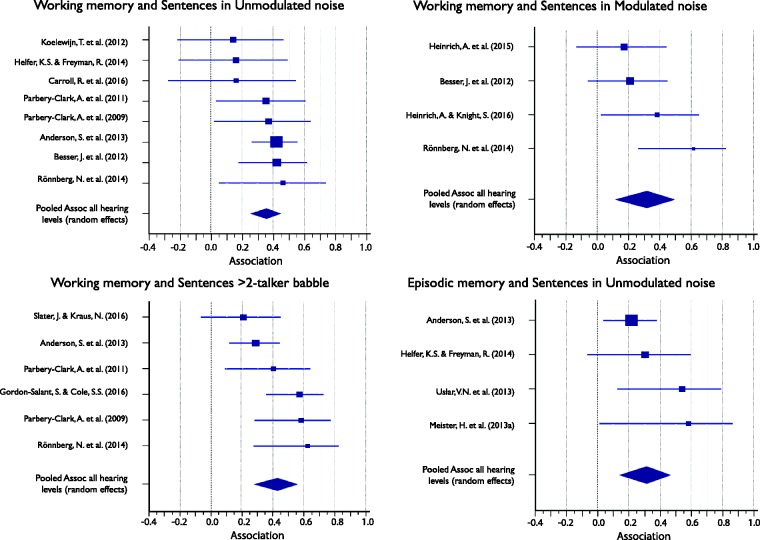
Forest plots showing the association between cognitive subdomains and speech-in-noise (SiN) speech and masker type combinations for all listeners. Marker sizes for individual studies (squares) are weighted on random-effects model weights. Whiskers represent 95% confidence interval. Pooled effects, calculated using a random-effects model, are shown as diamonds with the symbols extending to 95% confidence interval.

Associations ranged between .31 and .43, and all reached significance (Table 7). Figure 7 shows the Forest plots of the individual results contributing to, as well as the mean association of, each of the four meta-analyses. The Forest plots in Figure 7 indicate that while all contributing associations were positive, there was considerable variability in size and significance of individual associations contributing to each meta-analysis.

## Discussion

The association between cognitive performance and SiN perception has attracted increasing research interest over the past 20 years. However, at the individual study level, the outcomes that have been assessed are varied and inconsistent, and the findings have been mixed. In the current review, we have investigated three sources of variation: (a) a wide range of cognitive performance measures, (b) a wide range of SiN perception tests, and (c) variability in participants’ hearing thresholds. This research addressed these issues by categorizing cognitive measures into five cognitive domains and nine subdomains according to established cognitive theories. We also categorized the speech signal according to the lexical complexity of its target signal and the extent to which the background signal engages informational masking. Finally, we calculated effects for two participant groups; listeners with normal hearing to mild HL and those with normal hearing to moderate HL. Reported data were assessed in a series of formal meta-analyses where sufficient studies were available.

### General Association Between Cognitive Performance, SiN Perception, and HL

Collapsing across all cognitive domains and all SiN perception measures, there was an overall association of .31. Furthermore, the strength of the association did not vary depending upon HL groupings. This suggests that cognitive performance is associated with SiN perception and that this is independent of HL in the ranges examined.

### Attention

Alerting and orienting were expected to be generally important for SiN perception (Astheimer & Sanders, 2009; Heald & Nusbaum, 2014). Our review of the existing evidence shows that so far only a limited number of studies have investigated these relationships (two alerting and one orienting), and as a result, we were unable to perform a meta-analysis for this domain.

### Executive Processes

We hypothesized that executive processing may be linked to SiN perception and that the strength of the association may vary by subdomains. Only two of three executive processes subdomains (inhibitory control and WM) were reported in sufficient published studies to be (partially) assessed using meta-analyses.

Inhibitory control has previously been suggested to be important for SiN perception, particularly under informational masking conditions (Janse, 2012; Sommers & Danielson, 1999). It was assessed by six studies and was, with some combinations of SiN conditions, included in a meta-analysis. Overall inhibitory control showed a significant association with SiN perception of .34. Furthermore, the great majority of studies that assessed inhibitory control in connection with SiN perception used sentences as their target speech. Hence, it was not surprising that when the type of target speech was considered, the pooled association between sentences and inhibitory control was almost identical (.30) to the overall association. There was insufficient data to assess differences in association strength between inhibitory processes and different SiN masker types.

It has been suggested that WM is of general importance for SiN perception, regardless of specific target and masker types (Rönnberg et al., 2013) and perhaps particularly so for SiN perception tests that use sentence targets and more complex background maskers (e.g., Akeroyd, 2008). As many studies had included WM measures in their testing protocol, its role for various SiN measures could be evaluated in meta-analyses more thoroughly than the role of any other cognitive subdomain. The general association between WM and speech perception across all listeners was .28 with a slightly higher value for listeners with hearing in the range between normal to mild HL (.31) than listeners with hearing in the range between normal and moderate HL (.26). However, as the confidence intervals of both subgroups virtually overlapped, it was not possible to conclude that the association between WM and speech perception was moderated by (unaided) HL.

The speech target analysis showed similar and significant associations of .32 and .34 across both target stimulus categories for which enough data were available to test separately (i.e., words and sentences). When background masker types were considered separately for subcategories that provided enough data, significant correlations ranging between .26 and .39 were found for unmodulated noise, modulated noise, and >2-talker babble. It might be interesting to note that association strength appeared to increase with an increasing amount of informational masking in the background signal.

Finally, WM was one of the two cognitive subdomains (the other was episodic memory) that allowed the investigation of specific subdomain and listening situation combinations, with associations ranging between .32 and .43. While confidence intervals were again largely overlapping, it is interesting to note that mean associations appeared to be strongest when the background sound contained informational masking, and the target type was sentences.

### Memory, Intelligence, and Processing Speed

Episodic memory was expected to show an association with SiN perception particularly for more complex speech targets (Goldinger, 1996). We found that episodic memory showed an overall association with speech perception of .26 and that this association strength did not vary considerably where we could assess specific target speech signals or background maskers.

While there were sufficient studies assessing the association between speech perception and crystallized intelligence to conduct a meta-analysis, this was not the case for fluid intelligence. Crystallized intelligence has been suggested to be closely linked with SiN perception in terms of comprehension and lexical access (Schneider et al., 2016). When assessing target speech and masker background types separately, some interesting patterns emerged. When crystallized IQ was associated with SiN perception of any target speech type, masked by unmodulated noise, the pooled association was .13. However, when the target speech was sentences (collapsed across masker types), the association was numerically higher (.29). These data might suggest that the association between speech perception and crystallized IQ might be driven by the complexity of the target speech; however, there are insufficient data and studies to be confident in this conclusion.

Finally, we speculated that processing speed may be particularly important in situations with lexically complex speech targets due to an increase in processing required for memory retrieval (Gordon-Salant & Fitzgibbons, 2001; Wingfield et al., 1999). Overall, there was a significant association (.39) between SiN perception and processing speed when collapsing across all SiN categories. In terms of more fine-grained meta-analyses, SiN target type sentences showed a significant association with processing speed (.43).

### Patterns of Results in the Literature

This review highlights four important patterns in the published data, which only become evident when a large number of studies are simultaneously considered. First, it appears that the majority of associations between cognitive performance and SiN perception were of the magnitude of *r*≈.3, although the entire range of associations across all combinations was between .13 and .43. This was seen when collapsing data across cognitive domains and SiN categories, largely regardless of HL, and also when assessing specific cognitive subdomains, in particular inhibitory control, WM, and episodic memory. It is striking how little the association between SiN and cognitive performance differed across cognitive subdomains when the SiN target speech was sentences. As other types of target speech were comparatively rarely used, it is difficult to know whether a similar uniformity of associations would be seen for other types of target speech. Conversely, different combinations of cognitive subdomains and background maskers seem to vary more. Thus, being specific about the target and background signal as well as the tested cognitive subdomain and employing the full range of available stimuli may be a way to draw out further variability in association.

Second, it is interesting that although pooled associations were statistically significant, half of the associations from single studies that contributed to the meta-analyses (13 of 25) were not. This is particularly true for the cognitive subdomains of WM and episodic memory. In the case of WM, it also appears to be a particular issue for studies with listener groups in the range of normal hearing to moderate HL. Possibly, this result may highlight issues with low statistical power for individual studies (see the results of the risk of bias assessment—Table 1), so that the associations only become statistically significant when data are pooled.

The third key result of this review is that associations between SiN perception and many of the cognitive domains have so far been underinvestigated. Attention and fluid intelligence did not feature in enough included studies to warrant meta-analyses (*n* < 4). Even executive processes, which have been investigated in much greater detail, do not provide enough data to examine their role across the whole range of individual SiN target and background categories. For a comprehensive and detailed understanding of the relationship of cognition and SiN perception, a systematic investigation of the association between all cognitive subdomains and SiN target or masker types, even when we expect no significant correlations, would be informative. Negative or nonsignificant results are just as important as significant correlations because they allow us to understand the specificity of these results.

Finally, it is worth noting that when the moderating role of hearing ability was assessed, we found little difference in association between studies that included listeners with relatively better or poorer average unaided hearing thresholds, given the limited categorization we were able to apply.

## Limitations

There are some limitations of the current review. First, all cognitive tests were assigned to a specific cognitive domain by the authors to aid data categorization for assessment and reporting. However, it is recognized that any given cognitive test may actually assess a multitude of cognitive domains, and to different extents (e.g., Surprenant & Neath, 2009). We note that reassignment of complex cognitive tests to different respective cognitive domains or subdomains may lead to minor differences in the conclusions drawn from this research.

Second, cognitive domains were informed by multiple cognitive theories rather than on the basis of one specific unifying framework (although this could be viewed as a more informed and considered process than using a single theory). Third, we are limited in our conclusions by the available literature. For instance, we were not able to evaluate whether visual perception (perhaps indicating general differences in health or cognition) interacted with performance on cognitive tests (Scialfa, 2002) because virtually no studies measured this. Finally, the SiN categorization did not discriminate between adaptive and set level SNR paradigms, type of response set, different intelligibility levels or modes of signal presentation, and instead assumed that methodologies would engage cognitive processes in a similar way and to a similar extent. However, this may not be the case as suggested by the results of studies which have examined associations between cognition and nonadaptive SiN perception tests at multiple SNRs (Carroll, Warzybok, Kollmeier, & Ruigendijk, 2016; Heinrich & Knight, 2016; Tun & Wingfield, 1999) or adaptive SiN perception tests at multiple levels of intelligibility (Koelewijn, Zekveld, Festen, Rönnberg, & Kramer, 2012) within the same speech signal and masker type combination. In future studies, this assumption needs to be further examined, with investigations of associations between adaptive versus nonadaptive SiN perception tests and cognition being of potential interest to both basic scientists and clinical practitioners.

## Conclusion

Summarizing the results of this review, we conclude that (a) for cognitive performance and SiN perception, *r* = .3 appears to be the “magic number” for strength of association and (b) inhibitory control, WM, episodic memory, and processing speed are shown to be important for SiN perception, consistent with previous published evidence. These conclusions are based on literature which is selective in the specific measures and stimuli used, such that many alternative hypotheses have not yet been sufficiently assessed.

## Supplementary Material

Supplementary material

Supplementary material

Supplementary material
