# Supplementary material for: The Association Between Cognitive Performance and Speech-in-Noise Perception for Adult Listeners: A Systematic Literature Review and Meta-Analysis
Source: Trends Hear. 2017 Dec 14;21:2331216517744675. doi: 10.1177/2331216517744675 (PMC5734454; doi:10.1177/2331216517744675)
Supplement: Supplementary material [file supp_table_1_final_2.pdf]

Cognitive test by sub-domain	Description of test procedure
**1) ATTENTION**	
**Alerting**	
Integrated Visual and Auditory continuous performance test of attention (IVA+): Auditory Attention Quotient (AAQ) subtest (Sandford & Turner, 2004)	The numbers, 1 and 2, are presented aurally or visually. The task is to respond only presentations of '1' in either domain and to ignore presentation of the number '2'.
Test of Everyday Attention (TEA) task 7: Telephone search dual task (Robertson, Ward, Ridgeway, & Nimmo-Smith, 1994)	Visual search for key symbols in a telephone directory while simultaneously counting strings of tones presented by a tape recorder. The combined performance on sub-tests 6 and 7 gives a measure of divided attention - a 'dual task decrement'.
**Orienting**	
Test of Everyday Attention (TEA) task 6: Telephone search (Robertson et al., 1994)	Visual search for key symbols in a telephone directory page.
**2) EXECUTIVE PROCESSES**	
**Set-shifting**	
TMT B: Trail making test - B (Reitan, 1958)	The task is to connect, using a pencil, encircled numbers and letters in alternating order.
TMT B/A: Trail making test B/A (Reitan, 1958)	Ratio between parts TMT B and TMT A (B: A), B-A difference score may also be used - ratio and difference score proposed to attempt to partial out contribution of processing speed and motor speed. TMT A is described under Processing speed.
The connections test (Salthouse, 2000)	Similar to TMT B.
**Inhibitory control**	
Auditory Hayling task (Burgess & Shallice, 1996)	This test consists of two parts: firstly to give a verbal response to complete the final word of a sentence. The second part is to complete a sentence using a nonsense word, suppressing the predictable word.
Proactive interference (Kane & Engle, 2000)	Recall of lists of related or unrelated word lists (e.g. semantically or phonologically similar/dissimilar) after a distractor task (letter-number recall).
Simon task (Burle, van den Wildenberg, & Ridderinkhof, 2005)	Green and red circles are presented visually on a screen. The colored circles can appear on either side of the screen, however participants are given left and right arrow response keys which correspond to the color of the circle and not the position on the screen.
Stroop test - original (Stroop, 1935), computerized (Jesse & Janse, 2012)	The test contains two parts: firstly, a neutral condition, where a series of X's is printed in different color inks. Secondly, an incongruent condition, where a list of color names is printed in a different color ink to the word they represent, e.g., the word GREEN written in RED ink. The task in both conditions is to read out loud the color of the ink, and in the incongruent task to try to discount the meaning of the word. Stroop interference is calculated by subtracting the reading time neutral condition from the reading time of the incongruent condition.
Visual distraction test (May, 1999)	Identification of a target word based on commonalities between three visually presented cue words. Cue words are presented with and without additional (leading or misleading) distractor words.
**Working memory**	

Auditory working memory (Woodcock, McGrew, & Mather, 2001)	In lists of verbally presented words and numbers the task is to reorder the sequence, recalling the words first and the numbers second.
Backward digit recall (Wechsler, 1997)	Recall of verbally presented numbers in reverse serial order.
Digit ordering (Cooper, Sagar, Jordan, Harvey, & Sullivan, 1991)	Recall of verbally presented digit sequences in ascending order.
Letter-number sequence (re-ordering) (Gold, Carpenter, Randolph, Goldberg, & Weinberger, 1997; Wechsler, 2008)	Recall of verbally presented letter and number strings in ascending/alphabetical order.
Letter memory test (Morris & Jones, 1990)	Lists of consonants presented one at a time. The task is to recall the previous four letters (beginning once four letters have been presented) in correct serial order. List lengths vary between 5, 7, 9 and 11 letters.
Listening span test (Daneman & Carpenter, 1980)	Sentence lists are presented aurally. The task is to decide if the final word was predictable. A letter is presented visually with each sentence and the participant must recall the letter sequence in correct serial order after each sentence block.
Numbers reversed (Woodcock et al., 2001)	See backward digit recall
Operation span (Unsworth, Heitz, Schrock, & Engle, 2005)	Recall of lists of words and solution of simple math problems. First a math problem is displayed visually, e.g., Is (10+4)/2 = 8?, which participants read the problem out loud and judge its veracity with a button press of 'yes' or 'no'. Next a word is displayed for a short amount of time to be read aloud. The process repeats with word lists of increasing lengths. Finally, the task is to recall each word list in the correct serial order at the end of each trial.
Paced auditory serial addition test (Gronwall, 1977)	A random sequence of numbers (1-9) is presented aurally. The task is to add consecutive pairs of numbers such that each number is added to the number directing preceding it. The response is prompted when the number list has been fully presented.
Reading span test (Andersson, Lyxell, Ronnberg, & Spens, 2001; Besser, Koelewijn, Zekveld, Kramer, & Festen, 2013; Carroll et al., 2015; Daneman & Carpenter, 1980; Rönnberg, Lyxell, Arlinger, & Kinnefors, 1989)	The task is to read out loud lists of visually presented sentences and to remember the last word of each sentence for later recall. See (Conway et al., 2005) for review article on scoring methods.
Size comparison span (Sorqvist, Ljungberg, & Ljung, 2010)	Size-comparison sentences (e.g. is x bigger than y?) are presented visually, which are responded to with a 'yes' or 'no' response. After each comparison a to-be-remembered word is presented for later recall.
Visual letter monitoring (Gatehouse, 2003)	Identification of ten consonant-vowel-consonant words embedded within an 80-letter sequence displayed visually on a computer screen.
Colorado Assessment test: Visual working memory subtest (Davis & Keller, 1998)	A screen is displayed with eight boxes. The boxes light up individually to give a unique sequence. The task is to repeat back the sequence. The test has both forward and reverse conditions. The test is similar to the electronic Corsi block tapping test.
**3) MEMORY**	
**Episodic memory**	
Cognitive Spare Capacity Examination (CCSE) word recall (Jacobs, Bernhard, Delgado, & Strain, 1977)	Delayed word recall of four item lists.
Forward digit recall (Wechsler, 1997)	Recall of verbally presented numbers in correct serial order.

Letter-number sequence (serial recall) (Gold et al., 1997)	Recall of verbally presented sequences of letters and numbers in correct serial order.
Memory for words (Woodcock et al., 2001) Word list recall (Cervera, Soler, Dasi, & Ruiz, 2009; Schuchardt, Kunze, Grube, & Hasselhorn, 2006)	Recall of verbally presented unrelated word lists in correct serial order (similar to forward digit recall).
Verbal learning and memory test (Helmstaedter & Durwen, 1990) Word list memory (J. C. Morris et al., 1989)	Recall of as many words as possible from a verbally presented list. On subsequent trials the participant is reminded of any words missed from the previous trial - the trials repeat until all words are recalled.
**4) INTELLIGENCE**	
**Fluid IQ**	
Matrix reasoning (Wechsler, 1999)	Selection of one image, from a choice of five, to complete a matrix displaying images with a logical pattern.
**Crystalized IQ**	
Lexical decision test (Carroll, Warzybok, Kollmeier, & Ruigendijk, 2016)	Decision task as to whether or not visually presented monosyllabic words are real meaningful words or pseudo-words.
Mill Hill vocabulary scale (Raven, Raven, & Court, 1982)	Identification of the correct synonym of a target word in a six alternative multiple choice format.
Nelson-Denny reading test (Brown, Bennett, & Hanna, 1981)	Eight short passages are read followed by 36 multiple choice questions based on the eight passages, within 20 minutes.
Peabody vocabulary test (Bell, Lassiter, Matthews, & Hutchinson, 2001)	Visual presentation of four pictures and simultaneous auditory presentation of a target word. The task is to select the picture which best matches the target word.
Rhyme verification task (Johnston & McDermott, 1986)	A pair of words is displayed visually. The task is to decide if the word pair rhymes or not.
Verbal ability (Stenbäck, Hallgren, Lyxell, & Larsby, 2015)	Lists of five words are visually displayed. The task is to select two words in each list which are antonyms.
Vocabulary test (Wechsler, 1981)	Words are visually presented and the task is to give a definition of each word in turn.
Word vocabulary test (Snijders, Luteijn, & Verhage, 1983)	Similar to Mill Hill vocabulary scale, but in the Dutch language and a five-alternative multiple choice format.
Wortschatztest (Schmidt & Metzler, 1992)	Visual presentation of rows of words, each containing six words: five of which are pseudowords and one an existing word. The task is to select the one existing word in each row.
**5) PROCESSING SPEED**	
**Processing speed**	
Digit symbol substitution test (Wechsler, 1981, 1997)	The task is to copy symbols that are paired with geometric shapes or numbers in a set sequence.
Letter digit substitution test (Jolles, Houx, van Boxel, & Ponds, 1995; Wechsler, 1997)	Similar to the digit symbol substitution test.
Trail making test –A (TMT A) (Reitan, 1958)	The connection of, using a pencil, encircled numbers in numerical order displayed on a sheet of paper.

Supplementary Table 1.

Description of all cognitive tests used by the reviewed studies, categorized into cognitive domains and sub-domains
