# Supplementary material for: The Association Between Cognitive Performance and Speech-in-Noise Perception for Adult Listeners: A Systematic Literature Review and Meta-Analysis
Source: Trends Hear. 2017 Dec 14;21:2331216517744675. doi: 10.1177/2331216517744675 (PMC5734454; doi:10.1177/2331216517744675)
Supplement: Supplementary material [file supp_table_2_final.pdf]

| 2a | **Score: (✖, ?, ✓ or N/A)** |
|---|---|
| **Risk of Bias** | |
| 1. Did the authors include a sample size justification? | |
| 2. If any participant data is excluded from the analysis is a clear justification given? | |
| 3. Were all the outcome measures in the methods included in the results? | |
| 4. Were there any conflicts of interest? I.e., is the study funded or conducted by a body with vested interests in the results? | |

2b

✖ = High risk of bias (not enough information to make a judgement (Q1-3) or clear conflict of interest (Q4))
**?** = Unclear (incomplete information or not reported)
✓ = Low risk of bias (appropriate use and sufficient information (Q1-3) or no conflict of interest (Q4))
**N/A** = Not applicable (no participant data is excluded (Q2))

**Supplementary Table 2.**

*Checklist for risk of bias assessment. 2a: Questions assessing risk of bias; 2b: score key for all questions*
