# Supplementary material for: The Association Between Cognitive Performance and Speech-in-Noise Perception for Adult Listeners: A Systematic Literature Review and Meta-Analysis
Source: Trends Hear. 2017 Dec 14;21:2331216517744675. doi: 10.1177/2331216517744675 (PMC5734454; doi:10.1177/2331216517744675)
Supplement: Supplementary material [file supp_table_3_final.pdf]

**Study Demographics**	**Speech-in-noise Tests**	**Cognitive Tests**	**Sub-domain**	**Domain**
(Anderson et al., 2013)<br>N=120, 55-79 years, normal hearing-to-moderate hearing loss (HL)	Words in >2-talker babble (non-adaptive)<br><br>Sentences in >2-talker babble (non-adaptive)<br><br>Sentences in unmodulated noise (adaptive)	Auditory attention quotient of IVA+ (Sandford and Turner, 2004)	Alerting	Attention
		Memory for words (Woodcock et al., 2001)	Episodic memory	Memory
		Auditory working memory (Woodcock et al., 2001)	Working memory	Executive processes
(Besser et al., 2012)<br>N=55, 18-78 years, normal hearing-to-mild HL	Sentences in unmodulated noise (adaptive)<br><br>Sentences in modulated noise (adaptive)	Reading span test (Andersson et al., 2001)	Working memory	Executive processes
		Letter digit substitution test (Jolles et al., 1995)	Processing speed	Processing speed
(Carroll et al., 2016)<br>N=22, 18-35 years, normal hearing-to-mild HL	Sentences in unmodulated noise (non-adaptive)	Reading span test (Carroll et al., 2015)	Working memory	Executive processes
		The Lexical decision test (Carroll et al., 2016)	Crystalized IQ	Intelligence
		Wortschatztest (Schmidt and Metzler, 1992)	Crystalized IQ	Intelligence
		Peabody vocabulary test (Bell et al., 2001)	Crystalized IQ	Intelligence
(Cervera et al., 2009)<br>N=28, 19-25 & 55-65 years, normal hearing-to-moderate HL	Consonants in unmodulated noise (non-adaptive)	Word list recall (Cervera et al., 2009)	Episodic memory	Memory
		Digit ordering (Cooper et al., 1991)	Working memory	Executive processes
(Ellis and Rönnberg, 2014)<br>N=24, age 24 (mean) years (age range not available), normal hearing-to-mild HL	Sentences in ≤2-talker babble (non-adaptive)	Proactive interference (Kane and Engle, 2000)	Inhibitory control	Executive Processes
(Gordon-Salant et al., 2015)<br>N=24, 18-26 & 65-80 years, normal hearing-to-moderate HL	Words in >2-talker babble (non-adaptive)	Digit symbol substitution test (Wechsler, 1997)	Processing speed	Processing speed
(Gordon-Salant and Cole, 2016)<br>n=53, 18-25 & 61-75 years, normal hearing-to-mild HL	Words in >2-talker babble (adaptive)<br><br>Sentences in >2-talker babble (adaptive)	Listening span test (Daneman and Carpenter, 1980)	Working memory	Executive processes
		Paced auditory serial addition test (Rao et al., 1989)	Working memory	Executive processes

		Reading span test (Ronnberg et al., 1989)	Working memory	Executive processes
		Letter digit substitution test (Wechsler, 1997)	Processing speed	Processing speed
(Heinrich et al., 2015) N=44, 50-74 years, normal hearing-to-moderate HL	Sentences in modulated noise (non-adaptive)	Test of Everyday Attention subtest 6 (Robertson et al., 1994)	Orienting	Attention
		Test of Everyday Attention subtest 7 (Robertson et al., 1994)	Alerting	Attention
		Backward digit recall (Wechsler, 1997)	Working memory	Executive processes
		Visual letter monitoring (Gatehouse, 2003)	Working memory	Executive processes
		Reading span test (Daneman and Carpenter, 1980)	Working memory	Executive Processes
		Forward digit recall (Wechsler, 1997)	Episodic memory	Memory
		Matrix reasoning (Wechsler, 1999)	Fluid IQ	Intelligence
(Heinrich and Knight, 2016) N=30, 62-85 years, normal hearing-to-moderate HL	Words in modulated noise (non-adaptive)<br><br>Sentences in modulated noise (non-adaptive)	Stroop test (Stroop, 1935)	Inhibitory control	Executive processes
		Letter number sequencing (Wechsler, 1997)	Working memory	Executive processes
		Reading span test (Daneman and Carpenter, 1980)	Working memory	Executive processes
		Mill hill vocabulary scale (Raven et al., 1982)	Crystalized IQ	Intelligence
		Nelson-Denny reading test (Brown et al., 1981)	Crystalized IQ	Intelligence
(Helfer and Freyman, 2014) N=30, 45-85 years, normal hearing-to-moderate HL	Sentences in ≤2-talker babble (non-adaptive)<br><br>Sentences in unmodulated noise (non-adaptive)	Letter-number sequence serial recall (Gold et al., 1997)	Episodic memory	Memory
		Letter-number sequence re-ordering (Gold et al., 1997)	Working memory	Executive processes
		Connections test (Salthouse, 2000)	Set-shifting	Executive processes
		Stroop test (Jesse, 2012)	Inhibitory control	Executive processes
(Janse, 2012) N=39, 65-83 years, normal hearing-to-moderate HL	Phonemes in ≤2-talker babble (non-adaptive)	Stroop test (Stroop, 1935)	Inhibitory control	Executive processes
(Koelewijn et al., 2014) n=32, 31-76 years, normal hearing-to-moderate HL	Sentences in unmodulated noise (adaptive)<br><br>Sentences in ≤2-talker babble (adaptive)	Stroop test (Stroop, 1935)	Inhibitory control	Executive processes
		Listening span test (Daneman and Carpenter, 1980)	Working memory	Executive processes

		Size comparison span (Sorqvist et al., 2010)	Working memory	Executive processes
(Meister et al., 2013a) N=12, 58-79 years, normal hearing-to-mild HL	Sentences in unmodulated noise (adaptive)<br><br>Sentences in modulated noise (adaptive)<br><br>Sentences in >2-talker babble (adaptive)	Verbal learning and memory test (Helmstaedter and Durwen, 1990)	Episodic memory	Memory
(Meister et al., 2013b) N=26, 18-27 & 58-79 years, normal hearing-to-mild HL	Sentences in ≤2-talker babble (non-adaptive)	Verbal learning and memory test (Helmstaedter and Durwen, 1990)	Episodic memory	Memory
(Parbery-Clark et al., 2009) N=31, mean age 23±3 SD years (age range not available), normal hearing-to-mild HL	Sentence in unmodulated noise (adaptive)<br><br>Sentence in >2-talker babble (non-adaptive)	composite score of Auditory working memory and Numbers reversed (Woodcock et al., 2001)	Working memory	Executive processes
(Parbery-Clark et al., 2011) N=37, age 45-65 years, normal hearing-to-mild HL	Words in >2-talker babble (non-adaptive)<br><br>Sentence in unmodulated noise (adaptive)<br><br>Sentence in >2-talker babble (non-adaptive)	composite score of Auditory working memory and Numbers reversed (Woodcock et al., 2001)	Working memory	Executive processes
		Colorado Assessment test: Visual working memory subtest (Davis and Keller, 1998)	Working memory	Executive processes
(Rönnberg et al., 2014) N=20, 28-42 years, normal hearing-to-mild HL	Sentences in unmodulated (non-adaptive)<br><br>Sentences in modulated noise (non-adaptive)<br><br>Sentences in >2-talker babble (non-adaptive)	Reading span test (Rönnberg et al., 1989)	Working memory	Executive processes
		Letter memory test (Morris and Jones, 1990)	Working memory	Executive processes
(Slater and Kraus, 2016) N=54, 18-35 years, normal hearing-to-mild HL	Sentences in >2-talker babble (non-adaptive)	Auditory working memory (Woodcock et al., 2001)	Working memory	Executive processes
(Stenbäck et al., 2015) N=36, 18-22 & 61-79 years, normal hearing-to-mild HL	Sentences in modulated noise (adaptive)	Auditory Hayling task (Burgess and Shallice, 1996)	Inhibitory control	Executive processes
		Reading span test (Rönnberg et al., 1989)	Working memory	Executive processes
(Surprenant, 2007) N=75, 30-80 years, normal hearing-to-mild HL	Syllables in unmodulated noise (non-adaptive)	Rhyme verification task (Johnston and McDermott, 1986)	Crystalized IQ	Intelligence
		Operation span (Unsworth et al., 2005)	Working memory	Executive processes

(Tun and Wingfield, 1999) N=36, 18-22 & 61-79 years, normal hearing-to-moderate HL	Sentences in ≤2-talker babble (non-adaptive)<br><br>Sentences in >2-talker babble (non-adaptive)<br><br>Sentences in unmodulated noise (non-adaptive)	Vocabulary test (Wechsler, 1981)	Crystalized IQ	Intelligence
		Digit symbol substitution test (Wechsler, 1981)	Processing speed	Processing speed
(Uslar et al., 2013) N=20, mean age 24±2SD years (age range not available), normal hearing-to-mild HL	Sentences in unmodulated noise (adaptive)	word list recall (Schuchardt et al., 2006)	Episodic memory	Memory
(Veneman et al., 2013) N=15, 20-28 & 66-78 years, normal hearing-to-mild HL	Sentences in >2-talker babble (non-adaptive)	Visual distraction test (May, 1999)	Inhibitory control	Executive processes
(Zekveld et al., 2011) N=76, 19-31 & 46-73 years, normal hearing-to-mild HL	Sentences in unmodulated noise (adaptive)	Letter digit substitution test (Jolles et al., 1995)	Processing speed	Processing speed
		Word vocabulary test (Snijders et al., 1983)	Crystalized IQ	Intelligence
(Zekveld et al., 2014) N=24, age 22±2.8 SD years (age range not available), normal hearing-to-mild HL	Sentences in ≤2-talker babble (adaptive)	Reading span test (Besser et al., 2013)	Working memory	Executive processes
		Size comparison span (Sorqvist et al., 2010)	Working memory	Executive processes
		Letter memory test (Morris and Jones, 1990)	Working memory	Executive processes
		Trail making test B-A difference (Reitan, 1958)	Set-shifting	Executive processes

Supplementary Table 3.

Summary of included studies. Participant demographics include number of participants, age range, and Hearing Loss (HL) categorization. The identity of speech tests was characterized in terms of target stimulus (Phonemes, words, sentences) and masker (≤2-talker babble, >2-babble, modulated noise, unmodulated noise). Cognitive tests lists cognitive tests used in each study.
SiN and cognitive tests are only included in this table if they were eligible for analysis under the criteria of this review. Allocation of tests to cognitive domains and sub-domains is described in the main text. SD=standard deviation.
